# Probiotics for Mild Cognitive Impairment and Alzheimer’s Disease: A Systematic Review and Meta-Analysis

**DOI:** 10.3390/foods10071672

**Published:** 2021-07-20

**Authors:** Guangsu Zhu, Jianxin Zhao, Hao Zhang, Wei Chen, Gang Wang

**Affiliations:** 1 State Key Laboratory of Food Science and Technology, Jiangnan University, Wuxi 214122, China; su1994112@163.com (G.Z.); zhaojianxin@jiangnan.edu.cn (J.Z.); zhanghao@jiangnan.edu.cn (H.Z.); chenwei66@jiangnan.edu.cn (W.C.); 2 School of Food Science and Technology, Jiangnan University, Wuxi 214122, China; 3 (Yangzhou) Institute of Food Biotechnology, Jiangnan University, Yangzhou 225004, China; 4 National Engineering Research Center for Functional Food, Jiangnan University, Wuxi 214122, China; 5 Wuxi Translational Medicine Research Center, Jiangsu Translational Medicine Research Institute Wuxi Branch, Wuxi 214122, China

**Keywords:** probiotics, Alzheimer’s disease, mild cognitive impairment, cognition, meta-analysis

## Abstract

Accumulating evidence from animal studies supports the potential role of probiotics and prebiotics in alleviating neurodegenerative diseases. However, whether dietary supplementation with probiotics improves cognitive function in patients with Alzheimer’s disease (AD) or mild cognitive impairment (MCI) is unclear. We searched literature databases for relevant randomized control trials and compared the outcomes between control/placebo and intervention groups. The results of the included studies were meta-analyzed using a random-effects model, with standardized mean differences (SMDs) and 95% confidence intervals (CIs) calculated as summary statistics. We also performed a risk-of-bias assessment, sensitivity analysis and subgroup analysis. Among the 294 articles identified, eight articles involving 174 patients with AD and 446 with MCI were included in the qualitative synthesis and seven studies were meta-analyzed. Our analysis detected high between-group heterogeneity (SMD = 0.43, 95% CI −0.02–0.88, *p* < 0.0001, *I*^2^ = 86.4%) in cognitive function across the included studies. Subgroup analyses identified a significant effect of probiotics on cognitive function only in the studies involving people with MCI (*I*^2^ = 44%, *p* = 0.15 for heterogeneity, *p* = 0.0002 for overall effect). Our findings suggest that dietary supplementation with probiotics improves cognitive function, especially in people with MCI.

## 1. Introduction

Alzheimer’s disease (AD) is the most common neurodegenerative disease, hallmarks of which include amyloid plaques and neurofibrillary tangles [1]. Despite the existence of treatments that can alleviate these symptoms, no therapeutic approach has been proven to completely halt AD progression [2]. According to the 2020 World Alzheimer Report, the current annual healthcare cost for AD treatment is $1 trillion, which is predicted to double by 2030.

Due to the gradual nature of AD progression, it is imperative to explore and develop intervention strategies for early-stage AD [1]. In the past decade, an increasing number of randomized control trials (RCTs) have demonstrated promising findings regarding dietary interventions for AD, especially probiotic and prebiotic supplementation, which has been shown to delay AD progression [3,4]. Regulation of the gut–brain axis has been proposed as an evolving therapeutic approach for neurodegenerative disorders such as AD [5]. With the accumulation of knowledge regarding the changes in gut microbiota in patients with AD in recent years, research has increasingly focused on more specific ‘gut-microbiota-targeted’ intervention strategies for AD progression.

According to the consensus statement of the International Scientific Association for Probiotics and Prebiotics (ISAPP), probiotics are defined as ‘live microorganisms that, when administered in adequate amounts, confer a health benefit on the host’ [6]; the definition of a prebiotic is “a substrate that is selectively utilized by host microorganisms conferring a health benefit” [7]; and the definition of a synbiotic is “a mixture comprising live microorganisms and substrate(s) selectively utilized by host microorganisms that confers a health benefit on the host” [8]. Given their ability to modulate the structure and composition of the gut microbiota and impart health benefits, probiotics and prebiotics supplementation provide a novel approach for the prevention or treatment of certain diseases. In fact, several studies have provided compelling evidence supporting the neuroprotective effects of probiotics and prebiotics in neurological disorders [9,10,11].

Consistent with the burgeoning interest, several reviews of the efficacy of probiotic supplementation for neurological disorders/AD have been published, including one systematic review [12] and three meta-analyses [13,14,15]. Despite providing comprehensive evaluations of the early literature in this field and their importance for guiding clinical trials, these reviews have several limitations. For example, the meta-analyses yielded contradictory findings, with one reporting no ameliorative effect of probiotics on cognitive function [13] and the other two reporting improvement in cognitive function after the administration of probiotics [14,15]. These meta-analyses only included studies published before 2018 that investigated the beneficial effects of probiotics and prebiotics. We update the data by adding evidence from recently published studies [16,17,18], including one study that evaluated the protective effects of a synbiotic [16]. Furthermore, two of the three meta-analyses [14,15] included a study that combined mild cognitive impairment (MCI) and AD as a single outcome in their quantitative analyses, which might have yielded inaccurate findings. Notably, Jenifer et al. [13] only included three studies in their meta-analysis and did not assess publication bias, which might account for the high between-study heterogeneity reported. In addition, Cristofer et al. [15] did not include a subgroup analysis in their meta-analysis, despite observing high between-group heterogeneity. They also missed the most authoritative biomedical database, Embase, when searching for eligible literature.

To address these limitations, we conducted a systematic review and meta-analysis with more recently published clinical trials to evaluate the effects of probiotic and prebiotic supplementation on people with MCI and AD. To elucidate the possible beneficial effects of probiotics and prebiotics on cognitive function and neuroinflammation, we included a comparison of the treatment outcomes with outcomes in the control/placebo groups.

## 2. Materials and Methods

### 2.1. Search Strategy and Selection Criteria

This systematic review and meta-analysis is reported according to the Preferred Reporting Items for Systematic Reviews and Meta-Analyses (PRISMA) statement [19]. The PRISMA checklist is shown in Table S1. It has also been registered in the International Prospective Register of Systematic Reviews (Number CRD42020188410). We searched PubMed, Embase, Cochrane, Web of Science and ClinicalTrials.gov for relevant studies published in English between 1 July 1984 and 8 April 2021. The following MeSH terms and combined text were used to search the databases: Alzheimer’s disease, cognitive dysfunction, mild cognitive impairment, probiotics, prebiotics, synbiotics and randomized controlled trial. The search strategies used in specific databases are provided in Supplementary S1 We also manually searched the reference lists of the retrieved studies for relevant articles.

### 2.2. Eligibility Criteria

RCTs were eligible if they meet the PICO criteria (listed in Table 1). Studies were included if they (1) were randomized clinical trials (S) conducted in participants (P) with AD or MCI; (2) included an intervention (I) with probiotics, prebiotics or synbiotics; (3) compared the efficacy of the intervention with a control (C) or placebo; and (4) reported the main outcomes (O) of cognitive function (assessed using a rating scale) and gut microbiota diversity and composition. The additional outcomes reported could be changes in metabolic variables and inflammatory and oxidative stress biomarkers.

Studies were excluded if they (1) did not contain an AD or MCI cohort; (2) did not list the diagnostic criteria applied for AD or MCI; (3) were observational or retrospective; (4) did not evaluate an intervention strategy; and (5) contained duplicate study data.

The titles and/or abstracts of the retrieved studies were imported to EndNote X9 to assess for duplication. After eliminating the duplicate studies, all remaining studies were screened against the inclusion and exclusion criteria. The full texts of the studies that satisfied the inclusion criteria were retrieved. For the excluded studies, the reasons for exclusion were recorded. Two investigators (Guangsu Zhu and Gang Wang) independently reviewed the study titles and abstracts, and a third investigator (Jianxin Zhao) resolved disagreements.

### 2.3. Data Extraction

Data extraction was performed and crosschecked independently by two investigators (Guangsu Zhu and Gang Wang). Any disagreements were discussed with the third investigator (Jianxin Zhao). No grey literature sources were assessed in this review. The following data were extracted from the included studies: (1) general information (i.e., title, authors, publication year, trial registration number and country); (2) participant information (i.e., sample size, age, group, sex, participant demographics and baseline characteristics); (3) methodological information (i.e., study design, intervention and comparisons, treatment allocation, intervention description and trial period duration); and (4) result-related information (i.e., recruitment; main outcome data, such as cognitive function and gut microbiota composition and diversity; additional outcome data, such as metabolic variables, inflammatory biomarkers and oxidative stress; recorded adverse events; and study completion rates).

### 2.4. Risk-of-Bias Assessment

The risk of bias for each RCT was evaluated using the specific questions listed in the Cochrane risk-of-bias tool [20]. No study was excluded based on risk-of-bias assessment. Two investigators (Guangsu Zhu and Gang Wang) independently performed the assessment, and a third investigator (Jianxin Zhao) resolved disagreements.

### 2.5. Statistical Analysis

Meta-analysis was performed using the ‘metan’ command in Stata software version 14.0 (StataCorp, TX, USA), when two or more studies reported the same outcome. As cognitive function was measured using different rating scales in the included studies, its data were converted to standardized mean differences (SMDs) between the intervention and placebo groups. The effects of intervention in each included study were assessed by the changes from baseline. The SMDs and 95% confidence intervals (CIs) were calculated using a random-effects model.

Statistical heterogeneity across studies was assessed using the *I*^2^ statistic, with *I*^2^ values > 50% indicating moderate-to-high heterogeneity [21]. To explore the potential source of heterogeneity, subgroup analyses based on the disease severity, type of cognitive rating scale and number of intervention strains (single vs. multiple) were performed using Review Manager version 5.3 (Cochrane Collaboration, Oxford, UK).

To test the robustness of the results, a sensitivity analysis was performed using the leave-one-out method [22]. Publication bias was assessed by constructing a funnel plot with Begg’s and Egger’s tests.

## 3. Results

### 3.1. Literature Search and Study Selection

**Figure 1:**
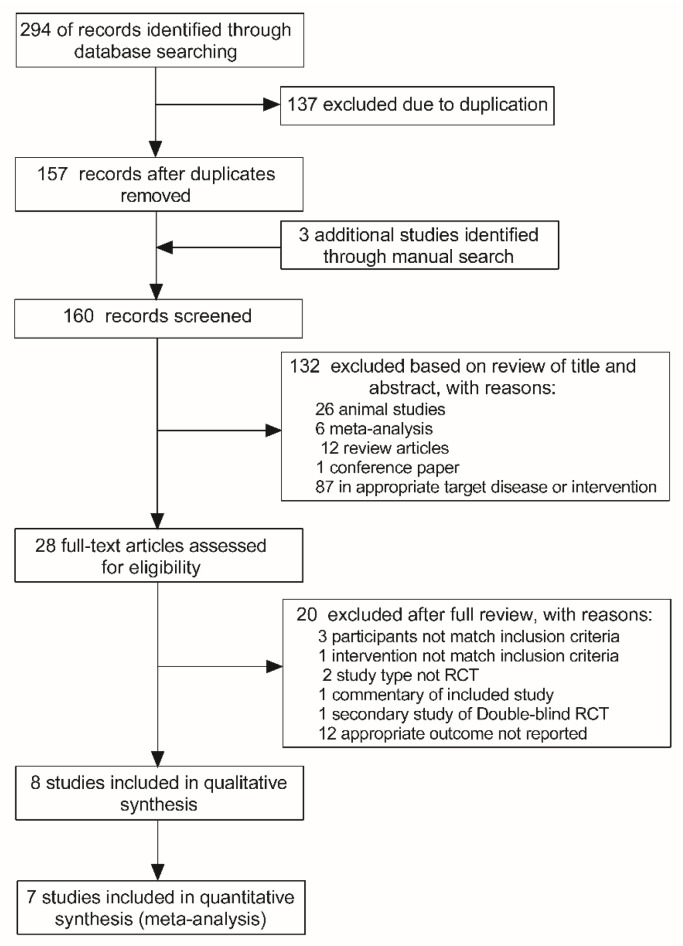
Flowchart illustrating the study selection process.

Two hundred and ninety-four articles were identified from the initial database screen and 157 remained after the removal of duplicate results (Figure 1). Then, three additional articles were added by manually searching the key articles included in reference list. Furthermore, 132 articles were excluded based on title and abstract screening and 28 full-text articles were assessed for eligibility. Subsequently, 20 articles were excluded for reasons detailed in the PRISMA flowchart (Figure 1). Ultimately, eight articles [16,17,18,23,24,25,26,27] were included in the qualitative synthesis (systematic review). One article [16] did not provide sufficient data for meta-analysis; therefore, only the remaining seven articles were included in the meta-analysis. Notably, one article [27] reported results from three independent studies using different cohort, which were regarded as three separate studies in the following quantitative analysis.

### 3.2. Characteristics of the Included Studies

The study characteristics of interest, including the country and type of study, population and diagnostic criteria, number and age of participants, proportion of female participants, intervention, species and dosage of probiotics/prebiotics, duration of the intervention, assessed outcomes and main findings, are presented in Table 2; Table 3.

As shown in Table 2, all eight of the included studies were published in the past 5 years. Among these, four studies [16,23,24,25] included patients with AD (174 subjects) and the other four [17,18,26,27] included people with MCI (446 subjects) aged 50–90 years. In seven of the eight studies, the proportion of female to male participants was higher, whereas one study did not report this data [25]. Three studies were conducted in Iran [23,24,25], one in Korea [26,27] and one in Japan [26,27] in 2019. In 2020, Sanborn et al. [17] investigated the effect of the well-known commercial strain Lactobacillus rhamnosus GG on cognitive function in the USA, and Ton et al. [16] evaluated the beneficial effects of traditional Kefir grain (synbiotic) supplementation in patients with AD in Brazil.

The intervention duration in most of the included studies [23,24,25,26,27] was 12 weeks, except for three studies. Two [16,17] of these three studies had an intervention duration of 90 days and one [18] had a longer duration of 16 weeks. Only one study [16] tested synbiotics as the dietary intervention. Four of the seven studies [17,18,26,27] tested a single strain, whereas the others [23,24,25] tested multiple strains of probiotics. Importantly, as presented in Table 3, all of the strains were of *Lactobacillus* and *Bifidobacterium*, the two most well-known and widely used probiotic genera. Six of the included studies [17,18,23,25,26,27] used encapsulated probiotics, whereas the other two [16,24] used pasteurized milk enriched with probiotics. In terms of dosage, all of the intervention group participants received a daily probiotic dose of 2 × 10^9^ CFU or more to ensure the activity of probiotics. Notably, most of the studies (except one [16]) applied a matched control/placebo intervention which was identical in appearance, taste and smell.

The primary outcome of all of the included studies was cognitive function, mainly evaluated using mini mental state examination (MMSE) scoring. The secondary outcomes assessed in most of the studies were nutritional status, oxidative stress, metabolic profiles and inflammation biomarkers. Notably, only Hwang et al. [26] reported the changes in gut microbiota.

With regard to the primary outcome, five studies [16,18,24,25,26] observed significant improvement in cognitive function after treatment with probiotics/synbiotics, whereas one study [23] found no significant change. Notably, mixed findings were reported in two studies, Kobayashi et al. [27] and Sanborn et al. [17]. Similarly, the secondary outcome results were also inconsistent across the studies. Notably, DW2009 administration significantly increased the concentration of serum brain-derived neurotrophic factor (*p* = 0.007) and enriched the relative abundance of *Lactobacillus* spp. (*p* = 0.045). However, no statistically significant change was observed in the relative abundances of *Bifidobacterium* spp. (*p* = 0.789) and *Clostridium* spp. (*p* = 0.936) after the interventions [26].

### 3.3. Risk-of-Bias Assessment

**Figure 2:**
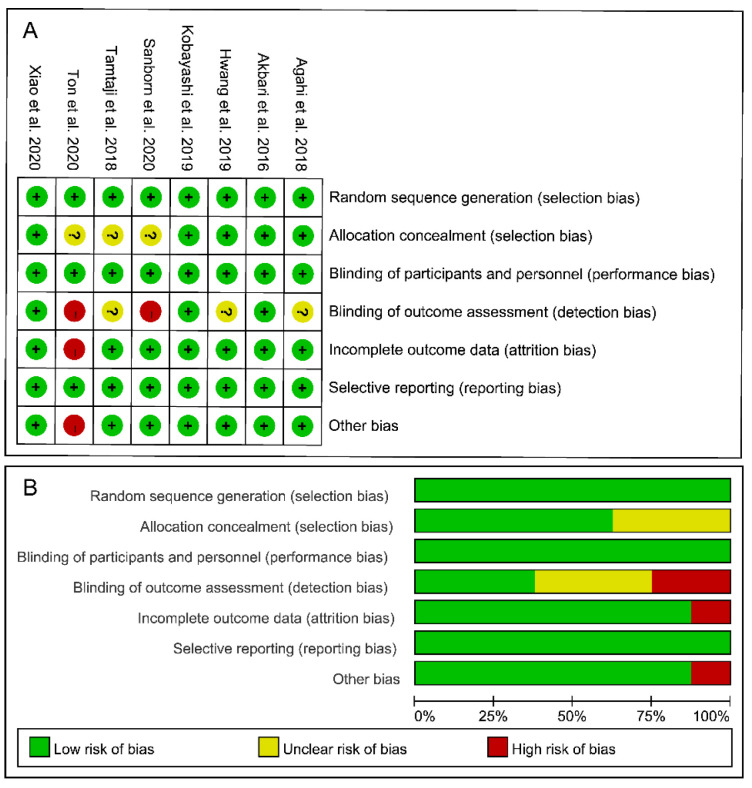
Risk of bias assessment for the included studies. (**A**) Risk of bias summary. (**B**) Risk of bias graph. Note: “+”: low risk, “?”: unclear risk, “-”: high risk.

The risk-of-bias assessment results for each included study are shown in Figure 2. Given that all eight of the included studies were double-blinded (participants and researchers) RCTs, they were all classified as having a low risk of selection and performance bias. However, 37.5% of the studies [23,25,26] did not describe the concealment of allocation and/or the blinding of outcome assessment. Two studies [16,17] did not mention whether the evaluators were blinded to the intervention allocation and were thus classified as having a high risk of detection bias. None of the included studies stopped their trials early and thus were all assigned a low risk of reporting bias. Notably, one study [16] only enrolled 13 participants and did not include a matched placebo group. As such, it was classified as having a high risk of attrition bias and other bias. Overall, based on the results for all six domains of the risk-of-bias tool, three studies [18,24,27] showed a low overall risk of bias, suggesting that these trials implemented strict procedures.

### 3.4. Main Findings of Meta-Analysis

**Figure 3:**
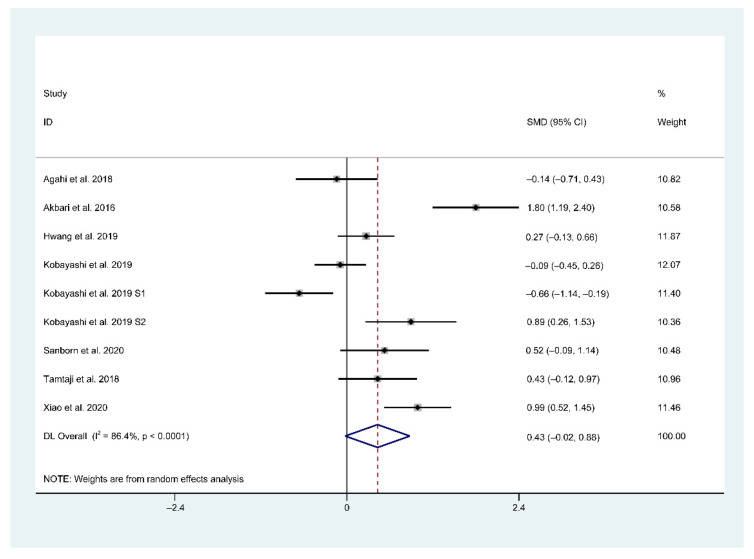
Forest plot of standardized mean difference (SMD) of probiotics on cognition. Weights were assigned according to the number of subjects and SD using STATA 14. A random-effect model was applied to the meta-analysis. The sizes of the data markers represent the weight of each study, and the diamond indicates the overall estimated effect. SMD, standardized mean difference.

In total, seven trials (included nine studies) provided sufficient data for a meta-analysis. In a pooled analysis of all included nine studies, a random-effects model was used when comparing the effects of probiotic and placebo treatments on cognition assessed using different rating scales. The results revealed high between-group heterogeneity (SMD = 0.43, 95% CI = −0.02–0.88, *p* < 0.0001, *I*^2^ = 86.4%) in the forest plot (Figure 3), indicating the inconsistency of results across the included studies.

### 3.5. Subgroup Analyses

**Figure 4:**
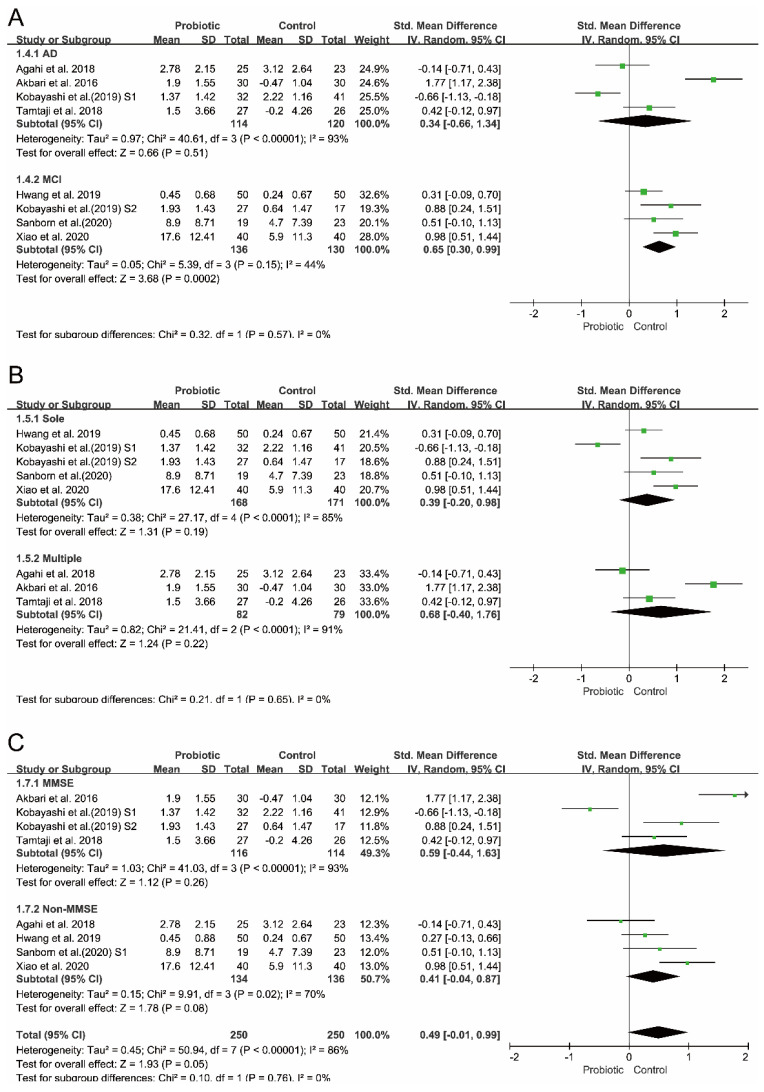
Subgroup analyses of the effect of probiotics on cognition. (**A**) Subgrouping by type of disease. (**B**) Subgrouping by strains of probiotics used in RCTs. (**C**) Subgrouping by cognitive rating scales.

To investigate the potential sources of between-study heterogeneity, three independent subgroup analyses were performed. As shown in Figure 4, the between-study heterogeneity (*p* = 0.57, *I*^2^ = 0%) decreased when the subgroups were stratified by disease type (AD vs. MCI). Similarly, heterogeneity (*p* = 0.55, *I*^2^ = 0%) in the random-effects model decreased when the subgroups were stratified by cognitive rating scales (MMSE vs. non-MMSE). Consistently, the analysis results of the subgroups stratified by ‘single vs. multiple’ strain intervention showed reduced heterogeneity (*p* = 0.65, *I*^2^ = 0%). However, none of these subgroup analysis results were statistically significant. A statistically significant effect on cognition was only observed in the subset of studies which included people with MCI (*I*^2^ = 44%, *p* = 0.15 for heterogeneity, *p* = 0.0002 for overall effect).

### 3.6. Publication Bias Assessment and Sensitivity Analysis

**Figure 5:**
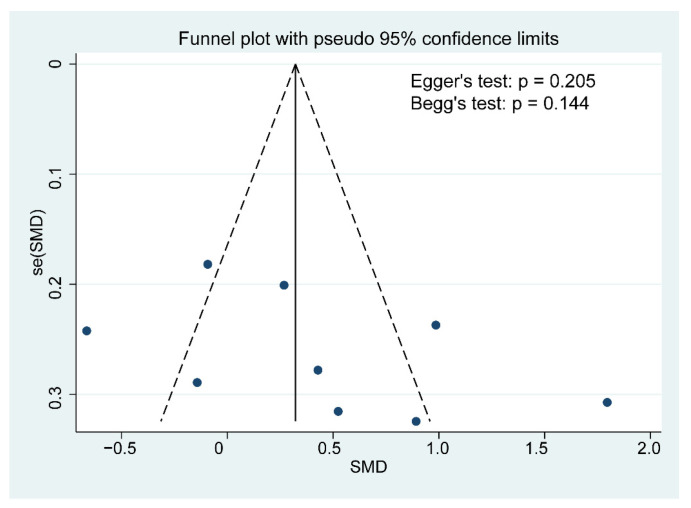
Funnel plot demonstrating publication bias in the meta-analyses. Each circle represents one study; and the vertical line indicates the weighted mean effect. The funnel plot asymmetry was assessed with Begg and Egger tests. A *p* value < 0.1 was defined as significant publication bias.

**Figure 6:**
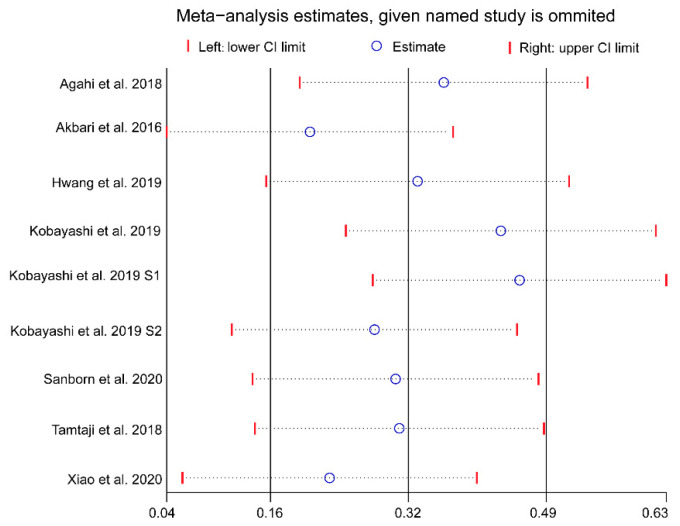
Results of sensitivity analysis using the leave-one-out method. Abbreviations: CI, confidence interval.

Although the funnel plot was slightly asymmetrical, no publication bias for cognitive function was detected based on the results of the Egger’s and Begg’s tests (Egger’s test: *p* = 0.205, Begg’s test: *p* = 0.144; Figure 5). A further sensitivity analysis revealed that the overall effects of probiotics on cognition was affected by one or more studies exclusions (Figure 6).

## 4. Discussion

In this meta-analysis, we include the most recent RCTs of probiotic and prebiotic supplementation for MCI and AD. Compared with placebo or control interventions, probiotic supplementation considerably improved cognitive function in the participants with MCI, but it only caused a modest cognitive improvement in those with AD. Furthermore, subgroup analyses conducted to explain the high heterogeneity across the included studies revealed that the extent of cognitive improvement is associated with the number of probiotic strains used (single or multiple), the dosage and duration of the intervention and the severity of the disease (AD or MCI). Collectively, these findings align with the previously reported neuroprotective effects of probiotics and prebiotics in neurological disorders [9,10,11].

In addition to cognitive improvement, probiotic supplementation altered the fecal microbiota structure and composition in AD patients. A recent study in a Chinese cohort found the diversity of fecal microbiota was significantly declined in AD patients; it also found reductions in the abundances of specific microbial communities to be associated with the disease severity of AD and MCI [28]. Similarly, a US study reported decreased fecal microbial diversity in patients with AD [29]. However, only one study [26] included in our review reported the changes of gut microbiome composition after probiotic administration. Given that microbiota-targeted interventions using probiotics/prebiotics can improve cognition through the microbiota–gut–brain axis, more attention should be paid to fecal microbiota analyses in future studies.

In the past decade, emerging studies have suggested that dietary probiotic intervention also play a beneficial effect in emotion, cognition, systemic and neural indices in disease states [30]. In addition to the clinical trials for AD and MCI reviewed in this study, several recent studies have evaluated the effects of specific probiotics on depression, anxiety and Parkinson’s disease. For example, in a randomized, double-blind, placebo-controlled clinical trial [25], daily probiotic administration for 12 weeks considerably improved the MDS-UPDRS scores in patients with Parkinson’s disease. Another recent study [10] reported that daily administration of probiotics led to a slight but significant change in symptoms of depression and anxiety, whereas prebiotic administration showed no effect.

Evidence from other investigations also support similar beneficial effects of probiotic administration on disorders of the gut–brain axis. For example, in one RCT [31], healthy volunteers randomly received a mixture of multiple probiotics or placebo for 4 weeks. Compared with the placebo, probiotic consumption significantly reduced reactivity to sad mood in the volunteers. Another study in physically healthy subjects also found that the consumption of a probiotic-containing yogurt for 3 weeks substantially improved mood [32]. An early study of irritable bowel syndrome also provided evidence of the beneficial systemic and immunological effects of probiotics [33]. Interestingly, only individuals with irritable bowel syndrome who received *Bifidobacterium infantis* 35,624 exhibited a normalization of the interleukin-10 to interleukin-12 ratio. These findings suggest that the widely acknowledged immunological benefits of probiotics are more strain-specific than previously thought [34].

Notably, the well-studied *Bifidobacterium* and *Lactobacillus* spp. are most frequently used as potential psychobiotics [35,36]. However, not all probiotics have psychobiotic potential. Thus, given the fact that the salutary effects of probiotics on AD are strain-specific, a more efficient probiotic screening method is warranted to develop effective probiotic strategies for AD.

According to the ISAPP definition of probiotics, consuming adequate amounts of probiotics can confer certain health benefits. However, the ISAPP does not specify the functional dose and frequency of probiotic supplementation. Over the past decade, organizations and agencies, such as the ISAPP [6], Health Canada [37], the World Gastroenterology Organization [38] and the Italian Ministry of Health (IMH) [39], have attempted to establish a recommended dosage of probiotics. To ensure safe and effective use of probiotics, the IMH has claimed that the minimum number of viable probiotic cells in food-based probiotics and dietary supplements should be 1 × 10^9^ CFU per day. Consistently, in our study, all of the included probiotic-treated studies consumed a daily probiotic dose of 1 × 10^9^ CFU or more. Despite the considerable research progress in the field of probiotic, determining the most effective dose of specific probiotic strains for different disease conditions remains a challenge. Given that certain probiotic strains have synergistic effects on the gut microbiota, multistrain probiotics that promise high efficacy should receive more attention in future studies.

Some limitations in the experimental design of the studies included in this systematic review must be acknowledged. First, some of the trials [16,23] had a small sample size, which might restrict the accuracy of the findings. Second, none of the included studies ruled out the interference of other dietary supplements, such as antibiotics, Mediterranean-style diets, other probiotics or fermented foods. Such dietary supplements may have a direct effect on the gut microbiota and metabolic profiles, which can in turn influence the gut–brain axis and related disorders. For instance, the results of a high-quality meta-analysis suggested that Mediterranean diets are negatively correlated with the risk of developing MCI and AD [40]. Therefore, such factors should be taken into consideration in future studies. Third, the included studies assessed cognitive function using a variety of rating scales. As the rating scales have different sensitivities and specificities for cognitive impairment, one or more gold-standard rating tools should be established to ensure the stability and reliability of results across studies. Fourth, to better distinguish patients with AD and without dementia, a commonly used clinical diagnosis criteria such as the NINCDS-ADRDA should be used. However, only four of our included studies [16,23,24,25] applied the NINCDS-ADRDA criteria. Fifth, none of the included studies ruled out the interference of other lifestyle interventions that can improve cognitive status and possibly even prevent cognitive impairment, dementia and AD [41], such as mental activities and exercise and specific multinutrient interventions [4]. Sixth, only two of the included studies [17,27] grouped the patients according to disease severity and evaluated the effects of probiotics on them separately. Evidence from previous researches have shown that the health effect of probiotics may differ among different diseases stages [26,27]. Consistently, our findings suggest that probiotic intervention at early stages of AD, such as MCI, could improve cognitive function and delay disease progression. Lastly, four of the included studies [17,23,24,25] did not report the adverse effects of probiotic administration. Although probiotics have a demonstrable history of safe use as dietary supplements, elderly people have decreased immunity and a high risk of serious adverse effects, such as gastrointestinal side effects, systemic infections and skin complications. Therefore, future studies are encouraged to report the adverse effects of probiotic supplementation [42]. By addressing all of these limitations in the design of future probiotic intervention-based studies, the beneficial effects of probiotics on AD could be more comprehensively and systematically clarified.

This systematic review has some noteworthy strengths. First, the systematic review and meta-analysis strictly followed the recommendations of the Cochrane handbook and the results were rigorously reported in accordance with the PRISMA statement. Second, we applied a random-effects model and performed sensitivity and subgroup analyses to adequately capture the heterogeneity among the study results. Third, we fully discussed the limitations of the experimental design of the included studies, addressing these methodological limitations may guide future researches.

Despite these strengths, our systematic review has some limitations. First, despite exhaustive literature searches, we might have missed some eligible studies. Second, the features of some included studies may have potential risk of bias due to commercial funding and defects in the experimental design. Third, the format of the data reported by some studies was not suitable for our analyses. Lastly, in terms of the high heterogeneity indicated, our findings should be interpreted with caution.

## 5. Conclusions

Collectively, the results of this meta-analysis indicate that probiotics, when supplemented at adequate amounts for 12 weeks or longer, may improve cognitive function in MCI or AD individuals. However, given the insufficient evidence from current RCTs, further work concerning long-period, large-scale RCTs are warranted to investigate the neuroprotective effects of probiotics in different stages of AD.

## Figures and Tables

**Table 1:** The PICOS criteria for inclusion and exclusion of studies.

Parameter	Inclusion Criteria	Exclusion Criteria
Participants	Individuals diagnosed with Alzheimer disease or mild cognitive impairment, based on any recognized diagnostic criteria.	People without cognitive impairment or combined with other types of dementia, e.g., vascular dementia, frontotemporal dementia.
Intervention	Probiotic or synbiotics, orally or enterally administered, with no restriction on strains, doses, frequency and duration of intervention.	Studies that compared or combined probiotics administration with drugs or other therapeutic interventions.
Comparison	Usual care, placebo, or other interventions without any probiotics/prebiotic/symbiotic supplementation.	N/A
Outcomes	Primary outcome: cognitive function, measured using an appropriate, validated cognitive test; gut microbiota diversity and composition. Secondary outcome: changes in metabolic variables and inflammatory and oxidative stress biomarkers.	Reported data could not be calculated based on the information in the article.
Study design	Randomized controlled trials (RCTs)	Case reports, review articles, systematic literature reviews, editorial pieces, comments, news and letters.

Note: N/A, not applicable.

**Table 2:** Characteristics of the included studies.

					Participants										
					Subjects (*n*)		Age (M & SD)		Gender (M/F)				Assessed Outcomes		
Study	Country	Study Type	Population	Diagnose Criteria	P	C	P	C	P	C	Intervention	Duration	Primary	Secondary	Main Findings
Agahi et al. (2018)	Iran	Double-blind RCT	48 AD individuals, aged 65–90 years	NINDS-ADRDA criteria	25	23	79.70 (1.72)	80.57 (1.79)	7/18	10/13	Probiotic, 2 capsules daily	12 weeks	TYM	TAC, GSH, MDA, IL-6, IL-10, TNF-α, 8-OHdG, NO, BMI	83.5% of the patients showed severe AD; no significant changes were detected both in cognitive and biochemical indications after probiotic supplementation.
Akbari et al. (2016)	Iran	Double-blind RCT	60 AD individuals, aged 60–90 years	NINDS-ADRDA criteria	30	30	77.67 (2.62)	82.00 (1.69)	6/24	6/24	Probiotic, 200 mL milk daily	12 weeks	MMSE	TAC, GSH, MDA, hs-CRP, NO, FPG, TG, TC, LDL, HDL, Insulin, HOMA-IR, HOMA-B, QUICKI, BMI	Cognitive function and some metabolic statuses were positively affected by probiotic supplementation in AD patients.
Hwang et al. (2019)	Korea	Double-blind RCT	100 MCI individuals, aged 55–85 years	Diagnostic and Statistical Manual of Mental Disorders, 5th edition (DSM-5)	50	50	68.0 (5.12)	69.2 (7.00)	20/30	14/36	Probiotic, 2 capsules daily	12 weeks	CNT (VLT, ACPT, DST)	BDNF, gut microbiota, TC, blood glucose, BMI, blood pressure, pulse rate, ALT, AST, ALP, Albumin, BUN, Uric acid, Creatinine	DW2009 supplementation can enhance cognitive function in MCI individuals.
Kobayashi et al. (2019)	Japan	Double-blind RCT	121 MCI individuals, aged 50–80 years	MMSE score, 22–27	61	60	61.5 (6.83)	61.6 (6.37)	30/31	30/30	Probiotic, 2 capsules daily	12 weeks	MMSE	RBANS, hs-CRP, blood pressure, pulse rate, BMI, TP, Alb, AST, ALT, LDH, ALP, γ-FTP, CREA, Uric acid, TC, TG, LDL-C, HDL-C, blood glucose, HbA1c	No significant difference was observed.
Reza et al. (2018)	Iran	Double-blind RCT	53 AD individuals, aged 65–90 years (mean age76.2–78.5)	NINDS-ADRDA criteria	27	26	76.2 (8.1)	78.5 (8.0)	Un	Un	Probiotic, 200 μg selenium + probiotic capsule daily	12 weeks	MMSE	TAC, GSH, hs-CRP, Insulin, HOMA-IR, QUICKI, TG, NO, FPG, MDA, LDL, VLDL, HDL, TC, HDL, BMI	Cognitive function and some metabolic profiles were improved by probiotic and selenium co-supplementation in AD patients.
Sanborn et al. (2020)	USA	Double-blind RCT	42 MCI individuals and 103 healthy individuals, aged 52–75 years	NIHTB	76	69	64.6 (5.58)	64.1 (5.32)	18/24	41/62	Probiotic, 2 capsules daily	90 days	NIH Toolbox [M1] scores (picture, flanker, case sort, list sort, pattern)	BMI, blood pressure	Probiotic supplementation improved the cognition score in subjects with cognitive impairment.
Xiao et al. (2020)	Japan	Double-blind RCT	80 MCI individuals, aged 50–79 years	lower RBANS score & MMSE score > 22	40	40	61.3 (7.7)	60.9 (6.9)	19/21	20/20	Probiotic, 2 capsules daily	16 weeks	RBANS (Immediate memory, Visuospatial/Constructional, Language, Attention, Delayed memory)	JMCIS score, blood pressure, pulse rate, BMI,	*B. breve* A1 supplementation significantly improved memory functions of suspected MCI subjects.
Ton et al. (2020)	Brazil	Double-blind RCT	13 AD individuals, aged 71–85 years	NINDS-ADRDA criteria	13	0	78.7 (3)	N/A	2/11	N/A	Synbiotic, 2 mL/kg.bw milk daily	90 days	MMSE	IL-6, IL-8, IL-1b, IL-12p70, TNF-α, IL-10, ROS, AOPP, MMP, p53, BMI, Cleaved PARP expression	Synbiotic supplementation improved cognitive deficits, systemic inflammation, oxidative stress, and blood cell damage in AD patients.

Note: RCT, randomized controlled trial; CFU, colony-forming units; AD, Alzheimer’s disease; MCI, mild cognitive impairment; NINCDS-ADRDA criteria, National Institute of Neurological and Communicative Disorders and Stroke (NINCDS) and the Alzheimer’s Disease and Related Disorders Association (ADRDA); MMSE, mini mental state examination; RBANS, repeatable battery for the assessment of neuropsychological status; BMI, body mass index; LDL, low-density lipoprotein cholesterol; HDL, high-density lipoprotein cholesterol; IL, Interleukin; TAC, total anti-oxidant capacity; GSH, total glutathione; MDA, malondialdehyde; hs-CRP, high-sensitivity C-reactive protein; NO, nitric oxide; DSM-5, Diagnostic and Statistical Manual of Mental Disorders, 5th edition; QUICKI, quantitative insulin sensitivity check index; VLT, verbal learning test; ACPT, auditory continuous performance test; DST, digit span test; NIH, National Institutes of Health; JMCIS, Japanese version of the MCI Screen; ROS, reactive oxygen species; AOPP, advanced oxidation protein products; MMP, mitochondrial membrane potential; PARP, poly (ADP-ribose) polymerase.

**Table 3:** Species and dosage of probiotic/prebiotic used in the included studies.

Study	Species of Probiotic/Prebiotic	Dosage
Agahi et al. (2018)	*L. fermentum*, *L. plantarum*, *B. lactis*, *L. acidophilus*, *B. bifidum*, *B. longum*, *B. bifidum*, *B. longum*	3 × 10^9^ CFU/day
Akbari et al. (2016)	*L. acidophilus*, *L. casei*, *B. bifidum*, *L. fermentum*	2 × 10^9^ CFU/g
Hwang et al. (2019)	*L. plantarum* C29 (DW2009)	1 × 10^10^ CFU/day
Kobayashi et al. (2019)	*B. breve* A1	2.0 × 10^10^ CFU/day
Reza et al. (2018)	*L. acidophilus*, *B. bifidum*, *B. longum*	2 × 10^9^ CFU/day
Sanborn et al. (2020)	*L. rhamnosus* GG	2 × 10^10^ CFU/day
Xiao et al. (2020)	*B. breve* A1	2 × 10^10^ CFU/day
Ton et al. (2020)	4% kefir grains containing *Acetobacter aceti*, *Acetobacter* spp., *L. delbrueckii*, *L. fermentum*, *L. fructivorans*, *Enterococcus faecium*, *Leuconostoc* spp., *L. kefiranofaciens*, *Candida famata* & *Candida krusei*	2 mL/kg.bw/day

Note: *L.* = *Lactobacillus*; *B.* = *Bifidobacterium*.

## Supplementary Materials

The following are available online at https://www.mdpi.com/article/10.3390/foods10071672/s1, Supplementary S1: Details of searching strategy and screening process, Table S1: PRISMA checklist.
