# Supplementary material for: Probiotics for Mild Cognitive Impairment and Alzheimer’s Disease: A Systematic Review and Meta-Analysis
Source: Foods. 2021 Jul 20;10(7):1672. doi: 10.3390/foods10071672 (PMC8303183; doi:10.3390/foods10071672)
Supplement: Supplementary file 1 [file foods-10-01672-s001.zip › Supplementary S1 Details of searching strategy and screening process.pdf]

Supplementary S1 Details of searching strategy and screening process

PubMed

Search strategy : (((("Alzheimer Disease"[Mesh]) OR ((((((((Alzheimer[All Fields]) OR (Alzheimer's[All Fields])) OR (Alzheimer's Disease[All Fields])) OR (Alzheimer Type Dementia[All Fields])) OR (Alzheimer Dementia[All Fields])) OR (Late Onset Alzheimer Disease[All Fields])) OR (Familial Alzheimer Disease[All Fields])) OR (Early Onset Alzheimer Disease[All Fields]))) OR (((((Cognitive Dysfunction[MeSH Terms]) OR (Cognitive Impairment[All Fields])) OR (Mild Cognitive Impairment[All Fields])) OR (Mild Cognitive Impairments[All Fields])) OR (Mild Neurocognitive Disorder[All Fields]))) AND ((((((((((Probiotics[MeSH Terms]) OR (probio*[All Fields])) OR (lactobacill*[All Fields])) OR (bifidobacter*[All Fields]))) OR (lactococcus*[All Fields])) OR (Saccharomyce*[All Fields])) OR (pediococcus[All Fields])) OR (streptococcus[All Fields])) OR (Lactic Acid Bacteria[All Fields])) OR (((((((((Prebiotics[MeSH Terms]) OR (Prebiotic[All Fields])) OR (inulin[All Fields])) OR (fructooligosaccharide[All Fields])) OR (fructo-oligosaccharide[All Fields])) OR (oligofructose[All Fields])) OR (lactulose[All Fields])) OR (Synbiotics[MeSH Terms])) OR (Synbiotic[All Fields])))) AND (((randomized controlled trial[Publication Type]) OR (randomized[Title/Abstract])) OR (placebo[Title/Abstract]))

Search results: 67 items

Web of Science

Search strategy:

Set	Results	Search history
# 4	101	#3 AND #2 AND #1
# 3	2659806	TS = (randomized controlled trial OR random OR placebo)
# 2	413803	TS = (probio* OR lactobacill* OR bifidobacter* OR lactococcus* OR Saccharomyce* OR pediococcus OR streptococcus OR Lactic Acid Bacteria OR prebio* OR inulin OR fructooligosaccharide OR fructo-oligosaccharide OR oligofructose OR lactulose OR synbio*)
# 1	365409	TS = (Alzheimer* OR Mild Cognitive Impairment OR Cognitive Dysfunction)

Search results: 101 items

Cochrane library

Search strategy:

ID	Search	Hits
#1	(Alzheimer):ti,ab,kw OR (Alzheimer's):ti,ab,kw OR (Alzheimer's Disease):ti,ab,kw OR (Alzheimer Type Dementia):ti,ab,kw OR (Alzheimer Dementia):ti,ab,kw OR (Late Onset Alzheimer Disease):ti,ab,kw OR (Familial Alzheimer Disease):ti,ab,kw OR (Early Onset Alzheimer Disease):ti,ab,kw	11366
#2	MeSH descriptor: [Alzheimer Disease] explode all trees	3473
#3	MeSH descriptor: [Cognitive Dysfunction] explode all trees	1675
#4	(Cognitive Impairment):ti,ab,kw OR (Mild Cognitive Impairment):ti,ab,kw OR (Mild Cognitive Impairments):ti,ab,kw OR (Mild Neurocognitive Disorder):ti,ab,kw	12113
#5	#2 OR #1 OR #3 OR #4	21862

#6	MeSH descriptor: [Probiotics] explode all trees	2080
#7	(probio*):ti,ab,kw OR (lactobacill*):ti,ab,kw OR (bifidobacter*):ti,ab,kw OR (lactococcus*):ti,ab,kw OR (Saccharomyce):ti,ab,kw OR (pediococcus):ti,ab,kw OR (streptococcus):ti,ab,kw OR (Lactic Acid Bacteria):ti,ab,kw	13957
#8	MeSH descriptor: [Prebiotics] explode all trees	293
#9	(Prebiotic):ti,ab,kw OR (inulin):ti,ab,kw OR (fructooligosaccharide):ti,ab,kw OR (fructo-oligosaccharide):ti,ab,kw OR (oligofructose):ti,ab,kw OR (lactulose):ti,ab,kw	3581
#10	MeSH descriptor: [Synbiotics] explode all trees	154
#11	(Synbiotic):ti,ab,kw	614
#12	#6 OR #7 OR #8 OR #9 OR #10 OR #11	16609
#13	#5 AND #12	71

Search results: 71 items

Embase

Search strategy:

No.	Query	Results
#10	#4 AND #8 AND #9	55
#9	'randomized controlled trial':ab,ti OR 'randomized':ab,ti OR 'placebo':ab,ti	965540
#8	#5 OR #6 OR #7	187521
#7	'synbiotic*':ab,ti	1975
#6	'prebio*':ab,ti OR 'inulin':ab,ti OR 'fructooligosaccharide':ab,ti OR 'fructo-oligosaccharide':ab,ti OR 'oligofructose':ab,ti OR 'lactulose':ab,ti	26754
#5	'probio*':ab,ti OR 'lactobacill*':ab,ti OR 'bifidobacter*':ab,ti OR 'lactococcus*':ab,ti OR 'saccharomyce':ab,ti OR 'pediococcus':ab,ti OR 'streptococcus':ab,ti OR 'lactic acid bacteria':ab,ti	166799
#4	#1 OR #2 OR #3	334429
#3	'mild cognitive impairment*':ab,ti OR 'cognitive dysfunction':ab,ti OR 'cognitive impairment':ab,ti OR 'mild neurocognitive disorder':ab,ti	114683
#2	'alzheimer':ab,ti OR 'alzheimer*':ab,ti OR 'alzheimer* disease':ab,ti OR 'alzheimer type dementia':ab,ti OR 'alzheimer dementia':ab,ti OR 'late onset alzheimer disease':ab,ti OR 'familial alzheimer disease':ab,ti OR 'early onset alzheimer disease':ab,ti	210312
#1	'alzheimer disease'/exp OR 'alzheimer disease'	215622

Search results: 55 items

Clinical Trials

Search strategy:

Condition or disease: Alzheimer disease or mild cognitive impairment

Intervention/treatment: probiotics or prebiotics or synbiotics

Search results：2 items

Title:	Study1: Intervention of Intestinal Microorganism in Mild Cognitive Impairment	Study2: Can Lifestyle Changes Reverse Early-Stage Alzheimer's Disease
Status:	Recruiting	Recruiting
Study Results:	No Results Available	No Results Available
Conditions:	Gut Microbiota<br>Mild Cognitive Impairment<br>Dementia, Alzheimer Type	Alzheimer Disease
Interventions:	Dietary Supplement: Probiotic supplemented intervention<br>Dietary Supplement: Placebo	Behavioral: Lifestyle medicine
Locations:	Department of Neurolgy, Xuanwu Hospital of Capital Medical University, Beijing, Beijing, China	Dean Ornish, M.D., Sausalito, California, United States
